# Grading urothelial carcinoma with probe-based confocal laser endomicroscopy during flexible cystoscopy

**DOI:** 10.1007/s00345-024-05122-x

**Published:** 2024-07-27

**Authors:** Ben-Max de Ruiter, Jan Erik Freund, C. Dilara Savci-Heijnink, Jons W. van Hattum, Theo M. de Reijke, Joyce Baard, Guido M. Kamphuis, D. Martijn de Bruin, Jorg R. Oddens

**Affiliations:** 1 Department of Urology, Amsterdam UMC, University of Amsterdam, Meibergdreef 9, Amsterdam, The Netherlands; 2 Cancer Center Amsterdam, Amsterdam, The Netherlands; 3 Department of Biomedical Engineering and Physics, Amsterdam UMC, University of Amsterdam, Meibergdreef 9, Amsterdam, The Netherlands; 4 Department of Pathology, UMC Utrecht, University of Utrecht, Heidelberglaan 100, Utrecht, The Netherlands; 5 Department of Pathology, Amsterdam UMC, University of Amsterdam, Meibergdreef 9, Amsterdam, The Netherlands

**Keywords:** Bladder cancer, Diagnostics, Confocal laser endomicroscopy, Cystoscopy

## Abstract

**Purpose:**

Urothelial bladder cancer (UCB) care requires frequent follow-up cystoscopy and surgery. Confocal laser endomicroscopy (CLE) is a probe-based optical technique that can provide real-time microscopic evaluation with the potential for outpatient grading of UCB. This study aims to investigate the diagnostic accuracy and interobserver variability for the grading of UCB with CLE during flexible cystoscopy (fCLE).

**Methods:**

Participants scheduled for transurethral resection of papillary bladder tumors were prospectively included for intra-operative fCLE. Exclusion criteria were flat lesions, fluorescein allergy or pregnancy. Two independent observers evaluated fCLE, classifying tumors as low- or high-grade urothelial carcinoma (LGUC/HGUC) or benign. Interobserver agreement was calculated with Cohens kappa (κ) and diagnostic accuracy with 2 × 2 tables. Histopathology was the reference test.

**Results:**

Histopathology of 34 lesions revealed 14 HGUC, 14 LGUC and 6 benign tumors. Diagnostic yield for fCLE was 80–85% with a κ of 0.75. Respectively, sensitivity, specificity, NPV and PPV were: for benign tumors 0–20%, 96–100%, unmeasureable-50% and 87%, for LGUC 57–64%, 41–58%, 44–53% and 54–69% and for HGUC 38–57%, 56–68%, 38–57% and 56–68%, with an interobserver agreement of κ 0.61.

**Conclusion:**

fCLE is currently insufficient to grade UCB.

**Supplementary Information:**

The online version contains supplementary material available at 10.1007/s00345-024-05122-x.

## Introduction

Bladder cancer ranks as 10th most prevalent cancer worldwide with over 500,000 new cases and over 200,000 attributable deaths yearly. Urothelial carcinoma of the bladder (UCB), which accounts for > 90% of all bladder cancers in high-income countries, is most common. [1] Approximately 75% of all newly diagnosed cases are non-muscle invasive bladder cancer (NMIBC). Despite local therapy of NMIBC, recurrence rates of up to 78% have been reported, demanding frequent and long-term cystoscopic follow-up after resection and adjuvant intravesical treatment. [2] Moreover, patients are exposed to high numbers of repeat transurethral resection of bladder tumors (TURBT) since outpatient cystoscopic assessment lacks the diagnostic ability for in-vivo histopathologic characterisation of suspicious bladder lesions. As such, the follow-up of NMIBC results in a substantial patient burden and healthcare costs [3]. Additionally, a Danish national cohort study reported that after histopathological evaluation of TURBT samples no neoplasia was identified in 36–53%, which contributes to a large number of excess surgeries. [4]

Confocal laser endomicroscopy (CLE), a probe-based optical imaging technique, seems to be a suitable tool for real-time assessment of suspicious bladder lesions during outpatient-based flexible cystoscopy. So far, CLE imaging yielded promising diagnostic accuracies for real-time identification and grading of UCB with the Cystoflex UHD-R probe (Mauna Kea, 50–65 μm imaging depth, 240 μm field of view, 1 mm lateral resolution) during rigid cystoscopy. The CystoflexUHD-R probe is, however, not compatible with flexible cystoscopes due to the 2.6 mm outer probe diameter of the ultra-high-definition lens system at the probe tip. Real-time CLE imaging during flexible cystoscopy is nevertheless feasible with the smaller CystoflexF probe (Mauna Kea Technologies) that lacks the ultra-high-definition lens system (outer probe of 1.0 mm). Despite having inferior imaging characteristics than the CystoflexUHD-R probe, the CystoflexF probe has identical imaging properties as the UroflexF probe (imaging depth of 40–70 μm, a field of view of 325 μm and a lateral resolution of 3.5 μm). The UroflexF probe yielded promising diagnostic accuracies for the identification and grading of urothelial carcinoma of the upper urinary tract. [5, 6] To our knowledge, the diagnostic accuracy of the CystoflexF/UroflexF probe for UCB has not yet been investigated. This study investigated the diagnostic yield, diagnostic accuracy and interobserver variability for the classification and grading of suspicious papillary bladder lesions with CLE during flexible cystoscopy using the CystoflexF. Moreover, a direct comparison with the diagnostic ability of the CystoflexUHD-R probe was performed.

## Material (patients) and methods

### Study design and patients

The CLETUR trial is a prospective pilot study of CLE to assess the diagnostic accuracy of probe-based CLE during flexible cystoscopy (fCLE) with the CystoflexF probe (Mauna Kea Technologies, France), in a paired study design (NCT05273593). This article describes the first objective of the CLETUR study. The results of this study were reported according to the STARD guidelines [7]. Patients were eligible and consecutively enrolled if planned for a TURBT for a suspicious papillary bladder lesions, as identified on outpatient cystoscopy. Patients with suspicion of Carcinoma in Situ (CIS) only were not eligible. Exclusion criteria were fluorescein allergy or pregnancy. Written informed consent was obtained from all participants. The study was approved by the institutional medical review board of each participating center and was conducted in accordance with the Guidelines for Good Clinical Practice (IRB 2019_197).

### Study procedures

In the operation room, a Foley catheter was inserted into the bladder for instillation of 200-400 ml of fluorescein 0.1% for 5 min to stain the extracellular matrix of the bladder mucosa. After removal of the catheter, the CystoflexF probe, connected to a low-power (488 nm) laser system (Cellvizio 100 series; Mauna Kea Technologies, Paris, France), was placed through the working channel of a 16Fr flexible cystoscope (Karl Storz, Tuttlingen, Germany) in direct perpendicular contact with the tumor of interest (TOI). Three subsequent 20-second recordings of three different sites on the TOI were obtained at 8–12 frames/s, displaying the cellular microarchitecture of the TOI. Then, a 26Fr continuous flow resectoscope with 30⁰ optics (Karl Storz, Tuttlingen, Germany) was introduced and the CystoflexUHD-R probe was inserted through the working channel to repeat the imaging protocol of the TOI as described above. Following CLE imaging, the TOI was resected according to the standard clinical protocol. Histopathological analysis was performed according to the standard clinical protocol by an experienced uropathologist (CDSH), blinded for CLE images. In case of UCB, grading was performed according to the WHO 2004/2016 for UCB.

### CLE image evaluation

In an offline setting, after a wash-out period of 12 weeks, the fCLE images were assessed by two experienced CLE observers (CDSH and JEF), who were blinded for clinical information. The fCLE images were assessed according to the previously validated CLE features by Liem et al. to differentiate between benign and malignant lesions. [5, 8, 9] In case of UCB, also histologic grade according to the WHO 2004/16 was classified.

After another wash-out period of at least 4 weeks, for exploratory purposes, observers where shown the WLC images after initial rating for a combined grading to assess clinical significance. Additionally both observers graded the CystoflexUHD-R images in an identical manner to compare their diagnostic accuracies. If both observers rated the CLE images as “insufficient data”, the CLE images were classified as non-diagnostic. These recordings were reported, but not included in analysis of diagnostic accuracy. Diagnostic yield was defined as the percentage of all CLE recordings classified as diagnostic. The assessment of fCLE was used as an index test and histopathology as reference test for the diagnostic accuracy calculations. Diagnostic accuracy was displayed as sensitivity, specificity, positive predictive value (PPV), and negative predictive value (NPV). The Cellvizio Viewer Software (Mauna Kea Technologies) was used for frame-by-frame analysis.

### Statistical analysis

The sample size for this study was determined in accordance with the IDEAL-D recommendations for exploratory studies, initially projecting an inclusion of 60 participants [10]. In light of the challenges posed by the COVID-19 pandemic, a re-evaluation of the sample size was conducted, resulting in an adjusted inclusion of 40. Baseline values were descriptively reported. The diagnostic accuracy, per observer and per CLE-probe were calculated with 2 × 2 tables. The interobserver variability for diagnostic yield and the diagnostic accuracy, were calculated with Cohen’s kappa.

All analyses were conducted in R version 4.03 (R Foundation for Statistical Computing, Vienna, Austria).

## Results

### Patient characteristics

**Fig. 1:**
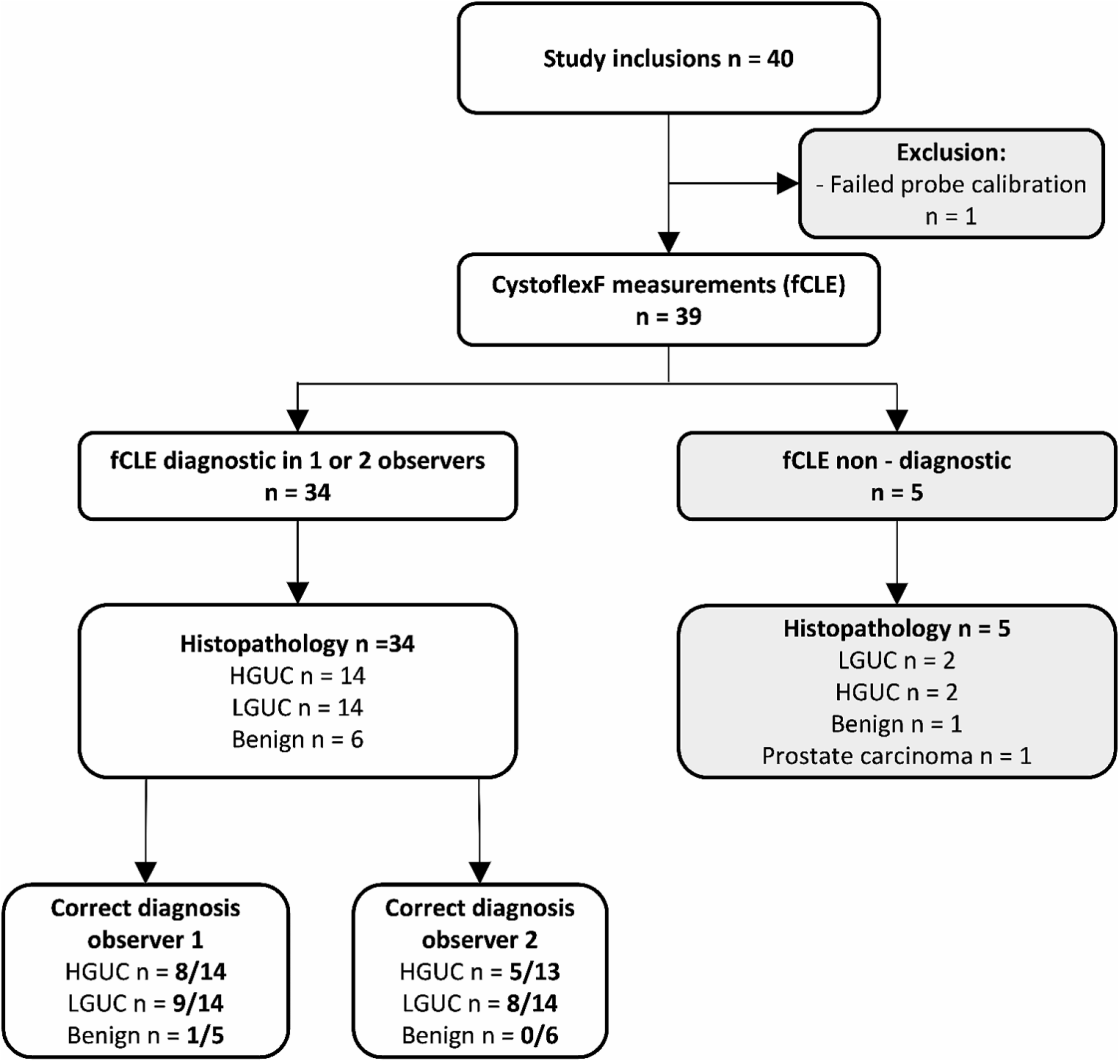
Enrollment overview; HGUC high grade urothelial cancer LGUC low grade urothelial cancer

40 measurements were performed from January 2020 until November 2021. Figure 1 displays the flow of participants. Baseline characteristics are displayed in Table 1. Benign lesions consisted of reactive changes (*n* = 2), papilloma (*n* = 2), nephrogenic adenoma (*n* = 1), inflammation (*n* = 1), and normal mucosa with enlarged vascular structures (*n* = 1).

**Table 1:** Patient and tumor characteristics

		Entire cohort (*n* = 40)		Diagnostic CystoflexF (*n* = 34)		Diagnostic CystoflexUHD-R (*n* = 31)	
**Age** *median [IQR]*		72 [62–76]		72 [65–76]		72 [60–74]	
		n	%	n	%	n	%
**Gender**	Male	33	83	30	88	25	81
**Prior UC**		15	38	13	38	11	35
**Number of tumors**	1 2 > 2	23 12 5	56 30 13	19 12 3	56 35 9	19 8 4	61 26 13
Tumor location*	Bladder Trigone Anterior wall Posterior wall Lateral Bladder dome	7 5 9 24 4	18 13 22 60 10	4 5 6 21 4	12 15 18 61 12	6 3 8 19 2	19 10 26 62 6
**Tumor size**	< 3 cm	35	88	30	88	26	84
**Histology**	LGUC HGUC Benign lesion PCa	16 16 7 1	40 40 18 2.5	14 14 6	41 41 18	11 15 5	35 48 16

*multiple locations were possible, UC = urothelial carcinoma, LGUC = low grade urothelial carcinoma, HGUC = high grade urothelial carcinoma, PCa = prostate carcinoma

### Diagnostic yield and accuracy

**Fig. 2:**
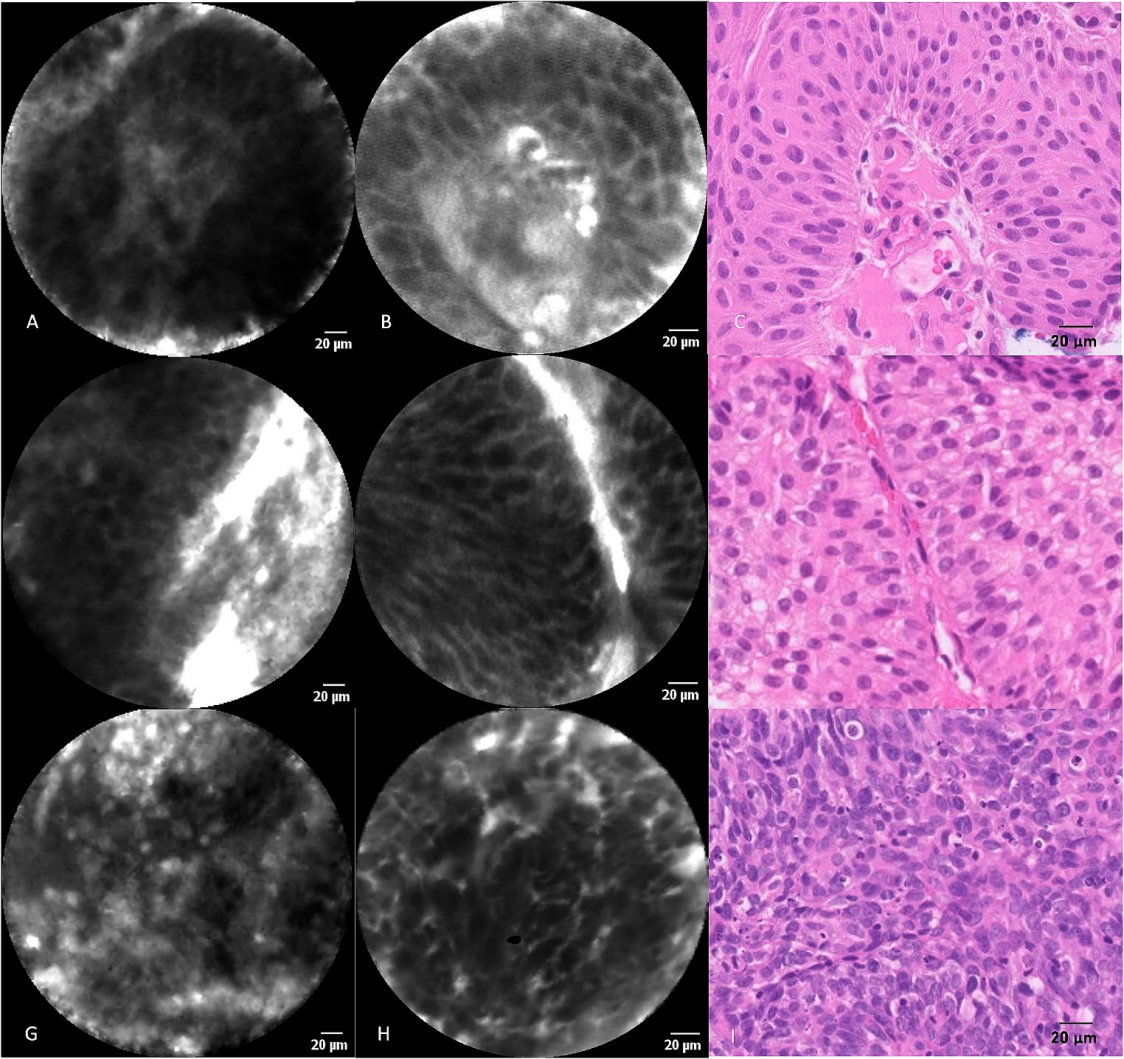
First column shows CystoflexF image, second column shows CystoflexUHD-R image, third column shows H&E image. A-F low grade papillary urothelial carcinoma A-C depicted with fibrovascular core. G-I high grade papillary urothelial carcinoma

The diagnostic yield per observer was 80–85% for CystoflexF measurements, with an interobserver agreement (k) of 0.75 (CI 0.7–0.93). Diagnostic yield per observer and histopathology, as well as corresponding 2 × 2 tables for both Cystoflex probes are available in the Supplementary Materials. Figure 2 displays the different resolutions of the CystoflexF and CystoflexUHD-R probe.

### CystoflexF

The diagnostic accuracy of the CystoflexF probe for UCB grading is displayed in Table 2 with a κ of 0.61 (CI 0.35–0.87). Adding WLC might slightly improve the diagnostic accuracy of fCLE (Supplementary Materials).

**Table 2:** Diagnostic accuracy of the CystoflexF and Cystoflex UHD-R probe for detection of UCB and benign tumors

		CystoflexF				CystoflexUHD-R			
		Sens. (CI)	Spec. (CI)	PPV (CI)	NPV (CI)	Sens. (CI)	Spec. (CI)	PPV (CI)	NPV (CI)
**Detection of LGUC**	**Observer 1**	64 (35–87)	58 (34–80)	53 (37–68)	69 (60–83)	64 (31–89)	68 (43–87)	54 (34–72)	76 (58–88)
	**Observer 2**	57 (29–82)	41 (18–67)	44 (30–59)	54 (34–73)	64 (31–89)	53 (29–76)	44 (29–60)	73 (51–86)
**Detection of HGUC**	**Observer 1**	57 (29–82)	68 (43–87)	57 (37–75)	68 (52–81)	64 (35–85)	63 (35–85)	60 (42–75)	67 (47–82)
	**Observer 2**	38 (13–68)	56 (31–78)	38 (21–60)	56 (41–69)	60 (32–84)	73 (45–92)	69 (47–85)	65 (48–79)
**Detection of benign disease**	**Observer 1**	20 (1–71)	96 82–100)	50 (7–93)	87 (81–91)	40 (5–85)	100 (86–100)	100 (16–100)	89 (80–94)
	**Observer 2**	0 (0–60)	100 (87–100)	-	87 (87–87)	20 (1–72)	100 (86–100)	100 (3-100)	86 (80–91)

HGUC = high grade urothelial carcinoma, LGUC = low grade urothelial carcinoma = Sens. = sensitivity, Spec = specificity, PPV = positive predictive value, NPV = negative predictive value,

### CystoflexUHD-R

The diagnostic accuracy of the CystoflexUHD-R is displayed in Table 2 for observer 1 and 2, respectively, with a κ of 0.58 (CI 0.32–0.85).

### CLE features CystoflexF

Presence of large vessels was highly predictive for the presence of UCB (LGUC or HGUC, k = 0.28 (CI 0.17–0.39)), as well as absent polarity of cells (k = 0.91 (CI 0.85–0.99)). Discriminating features for benign/LGUC versus HGUC were definition of cell borders, with a substantial agreement (k = 0.66 (CI 0.42–0.89)), and cohesiveness of cells, with a fair agreement (k = 0.24 (CI 0.12–0.45)). The distribution of histopathology per graded feature as proposed by Chang et al. is available in the supplementary materials. [9]

## Discussion

To our knowledge this is the first study that evaluated the diagnostic accuracy of fCLE for grading urothelial carcinoma of the bladder. Our findings show that CLE with the CystoflexF probe underperforms in terms of diagnostic accuracy and diagnostic yield in comparison to CLE with the CystoflexUHD-R probe during rigid cystoscopy.

When comparing the diagnostic accuracies of both techniques, the differences in NPV and PPV for fCLE versus standard CLE were most notable for benign and high grade disease. Our results using the Cystoflex UHD-R probe are in agreement with previous data that used the same probe during rigid cystoscopy. In these studies, sensitivity of 67–86% and specificity of 76–95% were reported for LGUC. For CLE-based detection of HGUC, sensitivity ranges of 67–95% were described [8, 11, 12]. These results are superior to our findings for fCLE. This discrepancy can be attributed to the inferior optic properties of the CystoflexF probe, characterized by lower resolution and a larger depth of the confocal plane. These differences in optic properties likely contribute to the variation in diagnostic accuracy observed between the two probes.

When evaluating the fCLE images, the presence of large vessels on CLE images were highly predictive for the presence of UCB, while the definition of cell borders and cohesiveness of cells were the most distinguishing features for LGUC versus HGUC and benign tumors. In our study, we have found that the imaging features for HGUC are consistent with the measurements observed in previous studies that utilized the CystoflexUHD-R. These findings suggest that the imaging characteristics of HGUC remain consistent across different measurement techniques, reinforcing the reliability and validity of our results. [8, 9]

A current limitation of fCLE is its diagnostic yield that arises from technical failures and insufficient image quality. In our study, even with urologists specifically trained for CLE, technical failure led to the exclusion of one patient in the CystoflexF group and six in the CystoflexUHD group. This aligns with findings from Liem et al., who reported one technical issue with the CystoflexUHD probe and labelled 11 out of 77 tumors as non-diagnostic [8]. The elevated rate of technical failure in the CystoflexUHD group could stem from its high data-processing demands or the use of two different probes. Additionally, our non-diagnostic rates were over 10% for the CystoflexF group and just below 10% for the CystoflexUHD-R group, consistent with Liem et al.‘s findings [8]. Notably, other prospective analyses have not commented on technical failures or non-diagnostic outcomes. [11, 13]

Interestingly, Freund et al. (2019) applied fCLE for grading upper tract urothelial carcinoma using the same optic properties and achieved a sensitivity and specificity of 77% and 63% for LGUC, respectively. [5] These values are higher than those reported in our study. This difference may be explained by the absence of benign tumors in their cohort.

The imaging characteristics acquired using fCLE in the bladder exhibit notable distinctions from previously documented features in the upper urinary tract. Particularly, the evaluation of cell borders was found to be insufficient for UTUC. This inconsistency can be attributed to various factors, related to the fixation of the probe, the perpendicular orientation of the probe against the TOI and histopathological architectural differences between bladder and ureter. Contrary to earlier observations in the upper tract, the CLE-criteria of cellular morphology and cell organization displayed variability and lacked predictive value in distinguishing between LGUC and HGUC in the bladder [5]. 

Strengths of this study are its prospective consecutive enrolment in a multicentre design, the comparative use of two CLE probes and the use of two blinded and experienced CLE observers. Limitations include the exclusion of patients with solely flat lesions. In addition, a single uropathologist was consulted for histopathological analysis, which is bivalent as it reduces inter-observer variability, despite the benefit of a consensus-based approach. [14]

Future research into CLE for UCB should focus on improving diagnostic accuracy of fCLE and reducing non-diagnostic measurements. Previous work into ex vivo combination of techniques such as photodynamic diagnosis and CLE did not appear feasible [15]. Recent work on CLE ex vivo has included the use of other fluorescent dyes or molecular staining, that could optimize specificity. [16, 17] Also recently, multispectral imaging has been shown to be feasible in vivo, combining multiple imaging modalities and complementing fluorescent signals [18]. In addition, the use of recurrent neural networks could overcome inter-observer variability. [12]

Based on the results in this study, future developments should focus on a more comprehensive scoring list of fCLE criteria including only highly predictive criteria, while also emphasizing the need for continuous improvement of the underlying technique to enhance accuracy and reliability.

## Conclusion

The diagnostic accuracy of fCLE for UCB is limited and inferior to standard CLE. Future research into fCLE should focus on improving the diagnostic accuracy and enhancing its technical reliability to improve the diagnostic yield.

## Electronic supplementary material

Below is the link to the electronic supplementary material.

Supplementary Material 1

Supplementary Material 2

## Abbreviations

CI: Confidence Interval
CLE: Confocal Laser Endomicroscopy
CIS: Carcinoma in Situ
fCLE: Flexible Probe Based Confocal Laser Endomicroscopy
HGUC: High-Grade Urothelial Carcinoma
IDEAL: Idea, Development, Exploration, Assessment, Long-term study
IRB: Institutional Review Board
κ: Kappa
LGUC: Low-Grade Urothelial Carcinoma
NMIBC: Non-Muscle Invasive Bladder Cancer
NPV: Negative Predictive Value
PCa: Prostate Carcinoma
PPV: Positive Predictive Value
Sens.: Sensitivity
Spec: Specificity
STARD: Standards for Reporting Diagnostic accuracy studies
TOI: Tumor Of Interest
TUR: Transurethral resection
TURBT: Transurethral Resection of a Bladder Tumor
UCB: Urothelial Carcinoma of the Bladder
UTUC: Upper Tract Urothelial Carcinoma
WHO: World Health Organization
