# Supplementary material for: Grading urothelial carcinoma with probe-based confocal laser endomicroscopy during flexible cystoscopy
Source: World J Urol. 2024 Jul 27;42(1):450. doi: 10.1007/s00345-024-05122-x (PMC11283388; doi:10.1007/s00345-024-05122-x)
Supplement: Supplementary file 1 — Supplementary Material 1 [file 345_2024_5122_MOESM1_ESM.docx]

Supplementary materials:

**Diagnostic Yield**

|  | **Diagnostic yield CystoflexF** | | | | **Diagnostic yield cystoflexUHD-R** | | | |
| --- | --- | --- | --- | --- | --- | --- | --- | --- |
|  | All (n=39) | Benign (n= 7) | LGUC (n=16) | HGUC (n=16) | All (n=34) | Benign (n=5) | LGUC (n=13) | HGUC (n=16) |
| Observer 1 | 85% | 71% | 94% | 87% | 91% | 100% | 100% | 94% |
| Observer 2 | 80% | 57% | 94% | 81% | 91% | 100% | 100% | 94% |
| k | 0.75 (0.7-0.93) | 0.09 (0.01-0.19) | 0 | 0.77 (0.54 – 0.96) | 1 | 1 | 1 | 1 |

**Observer 1 cystoflexF**

For high grade urothelial carcinoma

|  | Histopathology positive | Histopathology negative | Total |
| --- | --- | --- | --- |
| CLE positive | 8 | 6 | 14 |
| CLE negative | 6 | 13 | 19 |
| Total | 14 | 19 | 33 |

For low grade urothelial carcinoma

|  | Histopathology positive | Histopathology negative | Total |
| --- | --- | --- | --- |
| CLE positive | 9 | 8 | 17 |
| CLE negative | 5 | 11 | 16 |
| Total | 14 | 19 | 33 |

For benign

|  | Histopathology positive | Histopathology negative | Total |
| --- | --- | --- | --- |
| CLE positive | 1 | 1 | 2 |
| CLE negative | 4 | 27 | 31 |
| Total | 5 | 28 | 33 |

**For observer 2 CystoflexF**

For high grade urothelial carcinoma

|  | Histopathology positive | Histopathology negative | Total |
| --- | --- | --- | --- |
| CLE positive | 5 | 8 | 13 |
| CLE negative | 8 | 10 | 18 |
| Total | 13 | 18 | 31 |

For low grade urothelial carcinoma

|  | Histopathology positive | Histopathology negative | Total |
| --- | --- | --- | --- |
| CLE positive | 8 | 10 | 18 |
| CLE negative | 6 | 7 | 13 |
| Total | 14 | 17 | 31 |

For benign

|  | Histopathology positive | Histopathology negative | Total |
| --- | --- | --- | --- |
| CLE positive | 0 | 0 | 0 |
| CLE negative | 4 | 27 | 31 |
| Total | 4 | 27 | 31 |

**Observer 1 cystoflexF+WLC**

For high grade urothelial carcinoma

|  | Histopathology positive | Histopathology negative | Total |
| --- | --- | --- | --- |
| CLE positive | 10 | 5 | 15 |
| CLE negative | 4 | 13 | 17 |
| Total | 14 | 18 | 32 |

For low grade urothelial carcinoma

|  | Histopathology positive | Histopathology negative | Total |
| --- | --- | --- | --- |
| CLE positive | 9 | 8 | 17 |
| CLE negative | 4 | 11 | 15 |
| Total | 13 | 19 | 32 |

For benign

|  | Histopathology positive | Histopathology negative | Total |
| --- | --- | --- | --- |
| CLE positive | 0 | 0 | 0 |
| CLE negative | 4 | 28 | 32 |
| Total | 4 | 28 | 32 |

**For observer 2 CystoflexF+WLC**

For high grade urothelial carcinoma

|  | Histopathology positive | Histopathology negative | Total |
| --- | --- | --- | --- |
| CLE positive | 10 | 4 | 14 |
| CLE negative | 2 | 13 | 15 |
| Total | 12 | 17 | 29 |

For low grade urothelial carcinoma

|  | Histopathology positive | Histopathology negative | Total |
| --- | --- | --- | --- |
| CLE positive | 9 | 6 |  |
| CLE negative |  |  |  |
| Total |  |  |  |

For benign

|  | Histopathology positive | Histopathology negative | Total |
| --- | --- | --- | --- |
| CLE positive |  |  |  |
| CLE negative |  |  |  |
| Total |  |  |  |

**Observer 1 cystoflexUHD**

For high grade urothelial carcinoma

|  | Histopathology positive | Histopathology negative | Total |
| --- | --- | --- | --- |
| CLE positive | 9 | 6 | 15 |
| CLE negative | 5 | 10 | 15 |
| Total | 14 | 16 | 30 |

For low grade urothelial carcinoma

|  | Histopathology positive | Histopathology negative | Total |
| --- | --- | --- | --- |
| CLE positive | 7 | 6 | 13 |
| CLE negative | 4 | 13 | 17 |
| Total | 11 | 19 | 30 |

For benign

|  | Histopathology positive | Histopathology negative | Total |
| --- | --- | --- | --- |
| CLE positive | 2 | 0 | 2 |
| CLE negative | 3 | 25 | 28 |
| Total | 5 | 25 | 30 |

**For observer 2 CystoflexUHD**

For high grade urothelial carcinoma

|  | Histopathology positive | Histopathology negative | Total |
| --- | --- | --- | --- |
| CLE positive | 9 | 4 | 13 |
| CLE negative | 6 | 11 | 17 |
| Total | 15 | 15 | 30 |

For low grade urothelial carcinoma

|  | Histopathology positive | Histopathology negative | Total |
| --- | --- | --- | --- |
| CLE positive | 7 | 9 | 16 |
| CLE negative | 4 | 10 | 14 |
| Total | 11 | 19 | 30 |

For benign

|  | Histopathology positive | Histopathology negative | Total |
| --- | --- | --- | --- |
| CLE positive | 1 | 0 | 1 |
| CLE negative | 4 | 25 | 29 |
| Total | 5 | 25 | 30 |

Legend figure: Distribution of histopathology per graded feature LGUC low grade urothelial carcinoma HGUC high grade urothelial carcinoma

Diagnostic accuracy CystoflexF with WLC

|  | | **CystoflexF + WLC** | | | |
| --- | --- | --- | --- | --- | --- |
|  |  | Sens. | Spec. | NPV | PPV |
| **Detection of LGUC** | **Observer 1** | 69 (54- 75) | 55 (41 – 69) | 50 (30-83) | 73 (41- 90) |
|  | **Observer 2** | 69 (55 - 74) | 63 (35 - 85) | 56 (28 – 80) | 75 (43 – 91) |
|  | | | | | |
| **Detection of HGUC** | **Observer 1** | 71 (40 – 81) | 74 (51 – 90) | 67 (41-84) | 78 (60-96) |
|  | **Observer 2** | 85 | 74 (51 – 90) | 69 (41 – 86) | 88 (80-100) |
|  | | | | | |
| **Detection of benign disease** | **Observer 1** | 0 | 100 (10 – 100) | - | 82 (64 – 98) |
|  | **Observer 2** | 0 | 100 (10 – 100) | - | 81 (63 – 99) |
