# Supplementary figures and images for: Grading urothelial carcinoma with probe-based confocal laser endomicroscopy during flexible cystoscopy
Source: World J Urol. 2024 Jul 27;42(1):450. doi: 10.1007/s00345-024-05122-x (PMC11283388; doi:10.1007/s00345-024-05122-x)

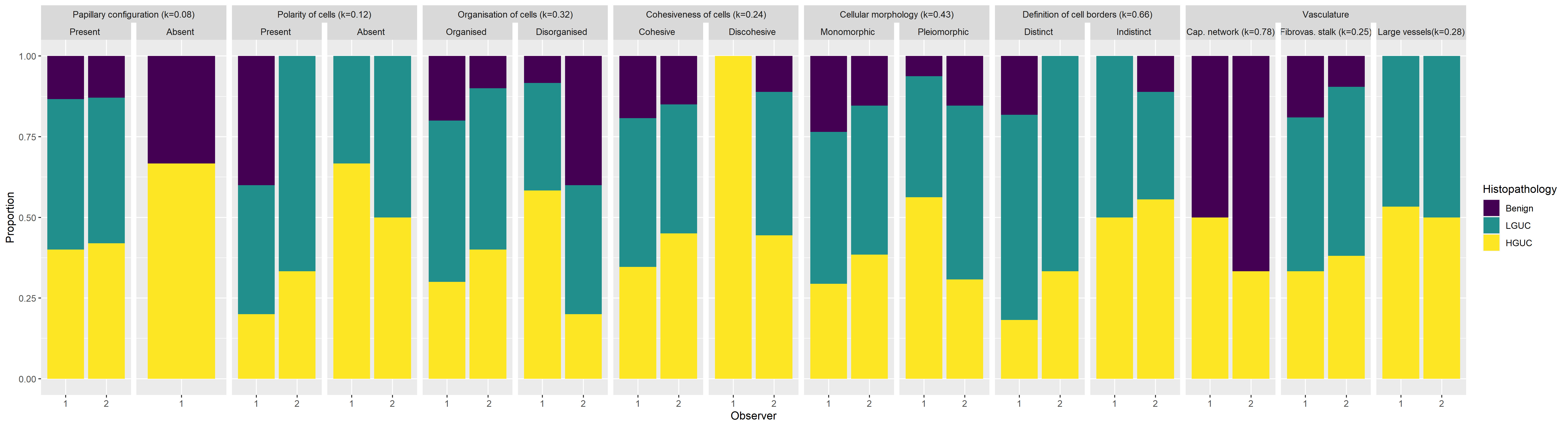

Supplement: Supplementary file 2 — Supplementary Material 2 [file 345_2024_5122_MOESM2_ESM.tiff]
